# Nilotinib-induced liver injury: A case report

**DOI:** 10.1097/MD.0000000000022061

**Published:** 2020-09-04

**Authors:** Youwen Tan, Yun Ye, Xingbei Zhou

**Affiliations:** Department of Hepatology, The Third Hospital of Zhenjiang Affiliated Jiangsu University, Zhenjiang, China.

**Keywords:** drug-induced liver injury, hyperbilirubinemia, nilotinib

## Abstract

**Introduction:**

Nilotinib is a selective inhibitor of the BCR-ABL tyrosine kinase receptor and is used in the management of chronic myelogenous leukemia (CML). Nilotinib therapy at high doses is associated with elevated serum bilirubin levels. If the serum bilirubin level exceeds 3 times the upper limit of normal, the recommendation is to either adjust nilotinib dosage or temporarily discontinue the treatment. However, it is unclear whether hyperbilirubinemia indicates obvious liver histology damage.

**Patient concerns:**

A 24-year-old man with confirmed CML was treated with nilotinib therapy and developed hyperbilirubinemia after the treatment. Although the first remission of the hyperbilirubinemia was achieved after dose adjustment, the hematological parameters deteriorated. Thus, we initiated an antineoplastic therapy (at the standard dose) until complete remission of the CML was achieved. The pathogenic mechanism of hyperbilirubinemia may be related to the inhibition of uridine diphosphate-glucuronosyltransferase (UGT1A1) activity. Liver histological analysis revealed no significant liver damage. In addition, the patient had no family history of hyperbilirubinemia and liver disease.

**Diagnosis:**

The patient was admitted to our hospital under the diagnosis of hyperbilirubinemia, and histopathology by liver biopsy showed no obvious damage. We also detected a UGT1A1 mutation [ex1 c.686C > A (p.Pro229Gln)] in the patient and his mother.

**Interventions:**

When the nilotinib dose was decreased to 300 mg daily, the total bilirubin (TBIL) level decreased to 30 to 50 μmol/L for 1 month. However, because the Bcr-Abl/Abl^IS^ ratio did not correspond to the major molecular response (MMR; <0.1%), the nilotinib dose was readjusted to 400 mg daily. One week later, the TBIL and indirect bilirubin levels increased to 89 and 79 μmol/L, respectively. The levels of alanine transaminase and other liver functional indicators were normal.

**Outcomes:**

A Naranjo Adverse Drug Reaction (ADR) Probability Scale score of 13 indicates that hyperbilirubinemia is attributed to ADR caused by nilotinib rather than by drug-induced liver injury.

**Conclusion:**

Although reducing the nilotinib dose can alleviate the occurrence of hyperbilirubinemia, the effect of MMR is also reduced. Treatment of CML without dose adjustment or discontinuation of nilotinib therapy may be more advantageous.

## 1 Introduction

Nilotinib is a selective inhibitor of the BCR-ABL tyrosine kinase receptor ^[1]^ and is used to treat Philadelphia chromosome, which is associated with chronic myelogenous leukemia (CML).^[2]^ Elevated serum aminotransferase levels are common in nilotinib therapy; however, only 4% to 9% of patients have been reported with levels 5 times higher than the upper limit of normal (ULN).^[3]^ Nilotinib therapy at high doses is also associated with elevated serum bilirubin level.^[4]^ If the alanine transaminase (ALT) or aspartate aminotransferase (AST) level increase significantly (i.e., if the ALT or AST level continues to be greater than 5 times the ULN, or the bilirubin level exceeds 3 times the ULN), the recommendation is to either adjust or temporarily discontinue the nilotinib dosage and initiate low-dose therapy.^[5]^ We recently encountered a case of CML treated with nilotinib therapy, which led to hyperbilirubinemia. Although the first remission of the hyperbilirubinemia was achieved after dose adjustment, the hematological parameters deteriorated. Thus, we initiated an antineoplastic therapy (standard dose) until complete remission of the CML was achieved. The pathogenic mechanism of hyperbilirubinemia may be related to the inhibition of uridine diphosphate-glucuronosyltransferase (UGT1A1) activity. Liver histological analysis revealed no significant liver damage.

## 2 Ethics Statement

Ethics statement is not applicable for case reports according to the medical ethics committee of the Third Hospital of Zhenjiang Affiliated Jiangsu University, but informed consent was obtained from the patient for publication of this case report and the accompanying images. The study was conducted in accordance with the Declaration of Helsinki.

## 3 Case report

A 24-year-old man was admitted to our hospital with a complaint of abdominal pain. His white blood cell count at admission was 361 × 10^9^/L. CML was suspected and later confirmed on the basis of the bone marrow biopsy result and the expression of Philadelphia chromosome (Ph+) on the cytogenetic study. First, 400 mg of imatinib mesylate (IM; Glivec, Novartis, Basel, Switzerland) daily was prescribed. The dose was reduced by 1 tablet daily when the patient first complained of leg ache. A rapid decline was observed in the platelet count from 231 × 10^9^/L to 54 × 10^9^/L in 20 days, and the dose was reduced by 2 tablets when the platelet count declined to 34 × 10^9^/L. After a month since the discontinuation of the nilotinib treatment, the Bcr-Abl/Abl ratio according to the international scale (IS) increased from 0.8% to 1%, and the nilotinib therapy (400 mg daily, twice a day; Tasigna; Novartis, Basel, Switzerland) was reinitiated.

**Figure 1:**
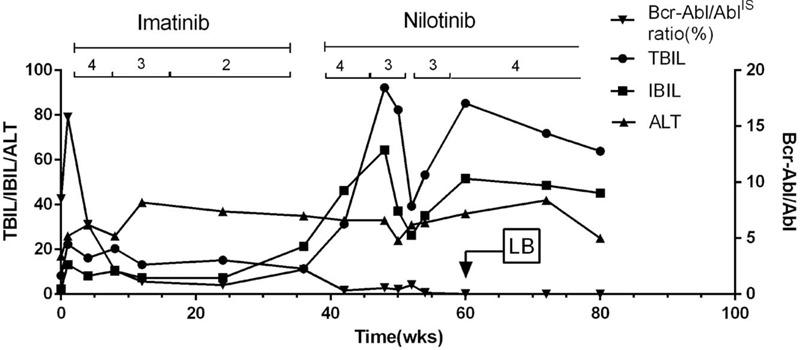
Course and evolution of the disease. 4 = 400 mg, 3 = 300 mg, 2 = 200 mg; LB, liver biopsy.

The findings from the liver function test performed before treatment were as follows: ALT, 32 U/L (normal range, 4–40 U/L); AST, 25 U/L (4–40 U/L); total bilirubin (TBIL), 12.6 μmol/L (3.4–21.2 μmol/L); direct bilirubin, 6.4 μmol/L (0.8–5 μmol/L); albumin, 3.5 g/dL (3.5–5.5 g/dL); prothrombin time, 12 s (9–14 s), alkaline phosphatase (ALP), 46 U/L (35–105 U/L); and γ-glutamyl transpeptidase, 32 U/L (normal, 5–36 U/L; Fig. 1).

**Figure 2:**
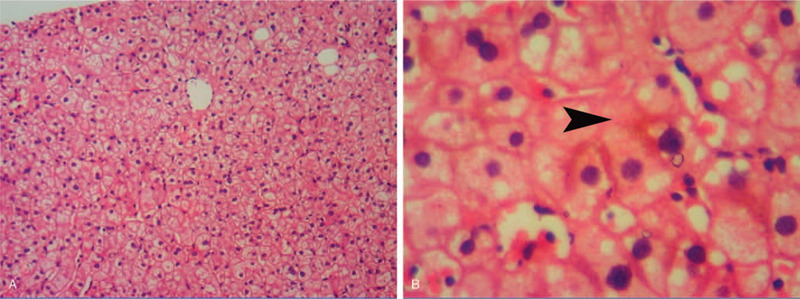
Microscopic features of the liver biopsy specimens. A: Normal hepatic lobule (hematoxylin and eosin staining, original magnification ×100). B: Bland cholestasis (hematoxylin and eosin staining, original magnification ×400).

After 4 weeks of nilotinib treatment, the TBIL level increased to 91 μmol/L. When the nilotinib dose was decreased to 300 mg daily, the TBIL level decreased to 32 μmol/L, which was maintained at approximately 30 to 50 μmol/L for 1 month. However, because the Bcr-Abl/Abl^IS^ ratio did not correspond to the major molecular response (MMR, <0.1%), the nilotinib dose was readjusted to 400 mg daily. One week later, the TBIL and indirect bilirubin (IBIL) levels increased to 89 and 79 μmol/L, respectively. The levels of ALT and the other liver functional indicators were normal. Liver biopsy was performed, and the histopathology showed no obvious liver damage (Fig. 2). The Bcr-Abl/Abl^IS^ ratio was <0.01% after 4 months of 400-mg nilotinib treatment, but the TBIL and IBIL levels were 60 to 80 and 40 to 60 μmol/L, respectively (Fig. 1).

**Figure 3:**
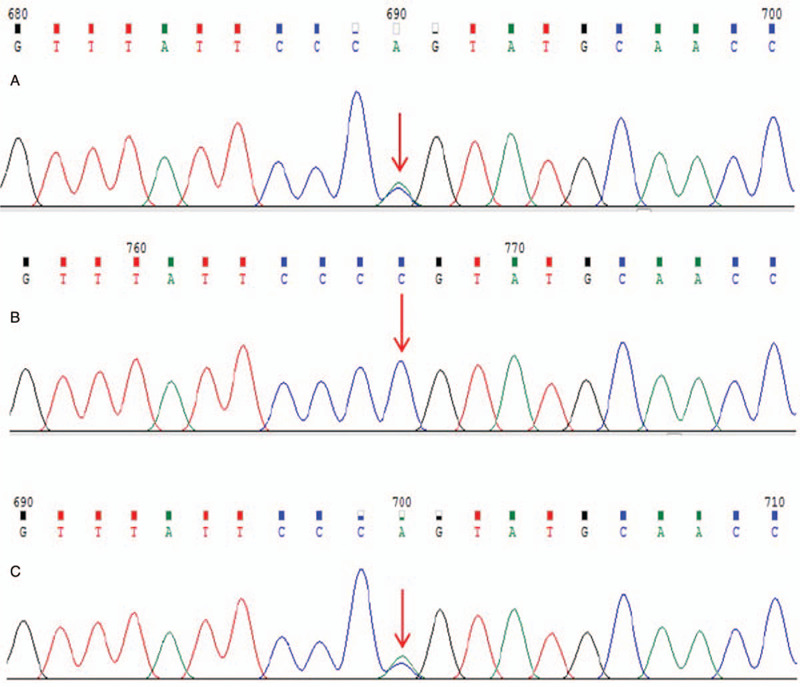
Subject: ex1 c.686C > A (p.Pro229Gln) Sanger sequencing map. A: Patient: had ex1 c.686C > A (p.Pro229Gln) gene mutation. B: Patient's father: had no ex1 c.686C > A (p.Pro229Gln) gene mutation. C: Patient's mother: had ex1 c.686C > A (p.Pro229Gln) gene mutation.

We also detected a UGT1A1 mutation [ex1 c.686C > A (p.Pro229Gln)] in the patient and his mother (Fig. 3). On the basis of the data from a database (https://www.ncbi.nlm.nih.gov/clinvar/), the gene had a missense mutation that caused the pathogenesis, and both the patient and his mother were asymptomatic carriers due to the heterozygous mutation. This finding showed that the hyperbilirubinemia observed in the patient was induced by the nilotinib administration and suggested that nilotinib therapy may inhibit UGT1A1 activity, including bilirubin glucuronidation. To this end, the liver histology and function showed normal manifestations. Moreover, viral hepatitis (A–E), Epstein–Barr virus, and cytomegalovirus infections, and hereditary metabolic liver diseases were ruled out.

## 4 Discussion

Imatinib is a revolutionary drug used in the treatment of CML.^[6]^ A single-drug therapy with imatinib can achieve complete cytogenetic responses and has attained an unprecedented overall survival rate.^[7,8]^ However, one-third of the patients who used 400-mg IM did not achieve satisfactory outcomes.^[9]^ Side effects such as progressive decrease in platelet count in patients restrict the use of IM in standard dose.^[10,11]^ In 2007, nilotinib was approved in the United States for the treatment of Philadelphia chromosome-positive CML,^[5]^ which is resistant or intolerant to previous treatments, including imatinib, thus opening up the possibility of using new treatment strategies for patients with CML.^[12,13]^

In a phase 1 dose-escalation study of nilotinib,^[14]^ 119 patients with imatinib-resistant CML were enrolled and received different nilotinib doses (50, 100, 200, 400, 600, 800, and 1200 mg). The results showed that the high-dose groups (17%, 6/35, 600–1200 mg) had a higher incidence of severely elevated bilirubin level than the 400-mg dose group (9%, 3/32).

In a randomized open-label multicenter study,^[15]^ nilotinib was shown to be a more potent inhibitor of BCR-ABL than imatinib. Moreover, the CCyR rates achieved in 12 months were significantly higher in the patients treated with nilotinib (300- or 400-mg dose) than in those treated with a similar imatinib dose. Of the patients in the 300- and 400-mg nilotinib dose groups, 53% (149/279) and 62% (171/277) had elevated TBIL levels, respectively, and 4% (10/279) and 8% (21/277) of the patients required dose adjustment and discontinuation of the nilotinib therapy, respectively.

Increased serum bilirubin level induced by nilotinib administration mostly causes indirect hyperbilirubinemia, as was observed in the present study, and is not associated with elevated serum enzyme levels or symptoms. No cases of nilotinib-induced liver failure have been reported.^[4,16]^ However, the existing literature recommends dose adjustment and discontinuation to reduce TBIL levels elevated to > 3 times the ULN.

In this case, the elevated TBIL level obviously improved through dose adjustment at the beginning of the TBIL level elevation, which also affected the response of the Bcr-Abl/Abl^IS^ ratio. Hua et al ^[17]^ reported that a 44-year-old man with CML developed metabolic acidosis within 10 hours of nilotinib administration, which rapidly progressed to multiorgan and hepatic failure (bilirubin, 4.4 mg/dL; ALT, 1031 U/L). Furthermore, the autopsy showed massive liver necrosis.

UGT1A1 is a key enzyme in bilirubin metabolism. Its activity can lead to indirect hyperbilirubinemia, which is associated with many hereditary hyperbilirubinemic conditions such as Gilbert or Crigler-Najjar syndrome.^[18]^ Although Gilbert syndrome is often described as a benign laboratory finding, it may alter drug metabolism by decreasing the ability to conjugate drugs.^[19]^ Nilotinib-induced hyperbilirubinemia has been reported to be mainly related to the decrease in UGT1A1 activity ^[20–23]^ (Table 1).

**Table 1:**
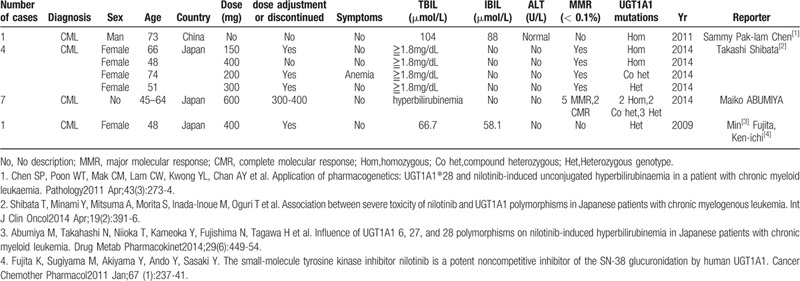
Nilotinib-induced hyperbilirubinemia associated with UGT1A1 activity.

Singer et al ^[24]^ reported that patients with CML who have high-risk mutations in UGT1A1 have an elevated risk of nilotinib-induced hyperbilirubinemia than patients with wild-type UGT1A1. Therefore, in their study, they recommended a reduced initial nilotinib dose for these patients to reduce the risk of hyperbilirubinemia. However, although reducing the nilotinib dose can alleviate the occurrence of hyperbilirubinemia, the effect of the MMR is also reduced without considering that high bilirubin level itself can cause slight damage to the human body.

Whether nilotinib-induced hyperbilirubinemia is a drug-induced liver injury (DILI) remains to be elucidated. An international DILI Expert Working Group of clinicians and scientists developed uniform clinical chemistry criteria for DILI as follows ^[25]^:

(1) ALT level of ≥5 times the ULN
(2) ALP levels of ≥2 times the ULN (particularly with accompanying elevations in 5′-nucleotidase or γ-glutamyl transpeptidase concentration in the absence of a known bone pathology underlying the increase in ALP level)
(3) ALT level of ≥3 times the ULN
(4) TBIL levels of ≥2 times the ULN. On the basis of these criteria, nilotinib-induced hyperbilirubinemia is not a DILI.

To assess the probability of adverse drug reactions (ADR), we used the Naranjo ADR Probability Scale ^[26]^ (Table 2). A score of 13 indicates that hyperbilirubinemia is attributed to ADR caused by nilotinib rather than to DILI.

**Table 2:**
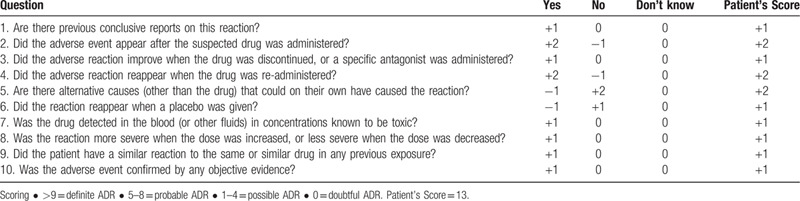
Naranjo Adverse Drug Reaction Probability Scale: Items and score.

In conclusion, nilotinib is a selective tyrosine kinase receptor inhibitor used in the treatment of CML. Hyperbilirubinemia is a common complication of nilotinib therapy, whose pathogenic mechanism may be related to the inhibition of UGT1A1 activity. In IBIL, hyperbilirubinemia (jaundice) has a less severe effect on patients. Considering that no significant liver damage was observed in the liver histological examination in the present case, treatment of CML without dose adjustment or discontinuation of nilotinib therapy may be more advantageous.

## Author contributions

**Investigation:** Yun Ye and Xingbei Zhou

**Supervision:** Youwen Tan

**Writing – original draft:** Youwen Tan and Yun Ye

**Writing – review & editing:** Youwen Tan
